# Bridging technology and care: integrating web-based PROMs in mental health services for refugees: a study on clinician training and technology adoption

**DOI:** 10.3389/fpsyg.2024.1355588

**Published:** 2024-06-04

**Authors:** Stine Bjerrum Moeller, Lotte Kring

**Affiliations:** ^1^Mental Health Services, Region of Southern Denmark, Department of Trauma and Torture Survivors, Vejle, Denmark; ^2^Department of Psychology, University of Southern Denmark, Danish Center of Psychotraumatology, Odense, Denmark

**Keywords:** technology in healthcare systems, PROM (patient-reported outcome measures), plan-do-study-act (PDSA), training program, thematic analysis (TA), focus group interviews, science and technology studies (STS), refugees

## Abstract

This study explores the integration of a web-based electronic database technology containing patient-reported outcome measures (PROMs) with electronic health records for refugees with PTSD, emphasizing the systematic inclusion of patient perspectives in clinical decision-making. Our research addresses the notable gap in literature regarding training clinicians for the competent integration of health information technology in healthcare. The training program developed aimed at equipping clinicians, particularly inexperienced with technology, to effectively utilize an electronic PROM system for collecting systematic patient information. Our study is set in the context of the Mental Health Services (MHS) in Denmark, focusing on a specialized clinic for treating trauma-affected refugees. The multidisciplinary team involved in this project reflects a wide range of healthcare professionals. The training program employed a variety of activities over nearly 2 years, adapting to feedback and aiming to engage clinicians in continuous improvement processes. Analyzing qualitative data with thematic analysis we interpreted that the training’s extended focus on discussion of the implementation process, with limited hands-on experience, potentially reinforced clinicians’ hesitations toward new technology, rather than reducing them. Clinicians prioritized immediate concerns over potential long-term benefits. Despite this, their approach reflects a strong commitment to patient welfare and careful evaluation of new practices. Notably, there were also positive engagements with the technology, highlighting its potential in patient care. This study concludes that the successful integration of technology in clinical settings hinges on its alignment with clinicians’ workflows, respect for their professional judgment, and clear benefits to patient care.

## 1 Introduction

Using health information technology in patient care is a global agenda. Despite its significance, there is a notable gap in literature regarding the training and education of clinical staff for competent integration of such technology in healthcare (Edirippulige and Armfield, 2017; Chike-Harris et al., 2021; Perle et al., 2022). Our study addresses this gap by integrating a web-based electronic database containing patient-reported outcome measures (PROMs) with electronic health records for refugees with Post-Traumatic Stress Disorder (PTSD), thereby systematically incorporating the patient perspective into routine clinical decision-making. Recognizing the critical need for clinicians to understand and value technology in patient care (Perle et al., 2013) this paper details the development and evaluation of a clinician training program focused on applying health information technology by using an electronic PROM system to collecting systematic patient information. In this paper, we explore the clinical setting, the pedagogical approach, the training content, and the impact of the training on clinicians’ confidence in using PROMs within a technological framework in their clinical practice.

Patient-reported outcome measures are standardized questionnaires which assess outcomes on the patient’s health conditions reported directly from the patient without interpretation of the patient’s response by a clinician or anyone else (Santana et al., 2015). The objective of routine PROM systems is to track patient needs and treatment results, guiding clinical practices and improving service quality. Additionally, they support research initiatives and aim to influence policy modifications and rehabilitative paradigm shifts, aligning services more closely with patient requirements (Clark et al., 2009; Gelkopf et al., 2022). If implemented with a simple design for collecting, analyzing, and applying the PROM data, PROMs have the potential to improve communication between clinicians and patients resulting in improved shared decision-making and health care (Foster et al., 2020; Gibbons et al., 2021). A recent Cochrane review corroborated that PROM feed-back improves communication between healthcare professional and patients (Gibbons et al., 2021). In Denmark, a Treatment and Research Integrated model using PROMs has been effectively implemented by pioneering scientists in a specialized clinic for refugee treatment within routine psychiatric services (Carlsson et al., 2014). Studies show various barriers to using PROMs in routine clinical practice, such as clinicians’ doubts about their clinical value, feeling that PROMs disrupt their practice, concerns about undermining their professional abilities, worries about managerial interference, fear of adversely affecting patients, skepticism of PROMs’ validity, unfamiliarity with them, time constraints, uncertainty in data interpretation for clinical relevance, and lack of integration into electronic health record systems (Santana et al., 2015; Stover et al., 2021; Gelkopf et al., 2022).

Despite its importance, the competency for using technology in psychiatry practice has been minimally addressed, highlighting a need for both advanced articulation and evidence-based pedagogical approaches for enhancement (Sunderji et al., 2015; Perle, 2020). Competence in using technology in psychiatry involves not only demonstrating knowledge but also skills (Hilty et al., 2017, 2020). While guidance on pedagogical methods for developing this competency is limited, a blend of didactic techniques, supervised clinical experience, case-based learning, and simulated assessments or role plays appears vital (Sunderji et al., 2015; Perle, 2020).

To effectively integrate a web-based database containing PROMs in clinical practice, clinicians must comprehend the purpose and value of this technological approach and develop adaptive attitudes toward its utility in clinical settings.

Acknowledging our clinicians’ novice status in integrating technology into patient care, we adopted a learning strategy sensitive to their potential uncertainty and unfamiliarity (Hilty et al., 2020). We used an extended time frame for our training to foster a culture of open discussion about uncertainties, consensus-building on the pros and cons of web-based technologies, experience sharing, and adapting to changes in clinical practices. In designing our educational approach, we aimed to root it in practice-based learning (Dornan et al., 2019), with the intention of engaging clinicians actively. The approach aims to foster engagement among clinicians by customizing their training according to individual needs, which in turn enhances their confidence in using the electronic database. The training structure was guided by the PDSA (plan-do-study-act) model for improving clinical quality and the principles of experiential learning theory (ELT) offered by Kolb (2014), emphasizing learning through the acquisition of abstract concepts applied flexibly in a range of situations. In Kolb’s theory, the impetus for the development of new concepts is provided by new experiences. The PDSA approach works well together with ELT, as it offers a “learning by doing” method that is yet both structured and theoretical. We intended for the combination of PDSA’s structured, hands-on methodology and ELT’s experiential learning to effectively support our clinicians’ development of competence. In the current study, we aimed to answer the following research questions:

1. *How do clinicians experience the clinician involvement in the training?*
2. *How do clinicians experience the training as helpful to prepare them to use the web-based electronic database with PROMs?*

## 2 Methodology

The study, a longitudinal study on the experiences of a training program, was part of a larger study on implementing a systematic collection of PROMs and sociodemographic data named The Danish Trauma Database (DTD) (Thøgersen et al., 2023). The PROM assessment battery includes information covering PTSD/CPTSD, anxiety, pain, and disability/general functioning. In addition, the structured clinical information includes semi-structured clinical interview covering PTSD/CPTSD, and screening for co-morbidity. The DTD initiative is a collaborative effort uniting six outpatient treatment clinics across Denmark, dedicated to supporting trauma-affected refugees. The DTD serves a dual purpose: it aids in clinical practice and supports research activities. In this study, we used qualitative data to explore clinicians’ experiences of our training to prepare them to use the electronic database in their everyday clinical practice.

### 2.1 Study setting and participants

The study was set within the Danish healthcare system, where five regions—North, South, Central, Capital, and Zealand—are responsible for hospitals, general practitioners (GPs), and mental healthcare services. Each region includes specialized clinics for treating trauma-affected refugees as part of their mental healthcare offerings. This study focuses on the Department for Survivors of Trauma and Torture in the South Region, which treats about 550 refugees annually. Patients are referred to the trauma clinic by hospitals, GPs, or private psychiatrists for the assessment and treatment of traumatic distress. Criteria for treatment at the clinic include being 18 years or older, having a refugee background, experiencing trauma-related mental health issues, and holding a permanent or temporary residence permit in Denmark. Those not eligible for treatment at the clinic or presenting with acute psychosis, a high risk of suicide, or severe drug abuse are directed to other appropriate primary care services, coordinated with GPs, or to different treatments within the clinic if it caters to other patient groups. Before the clinic adopted the electronic database, patient health information was collected and utilized in an unsystematic manner throughout patients’ treatment, with clinicians summarizing the data in the patients’ electronic health records.

The multidisciplinary team participating in the training program, aimed at integrating the electronic database into clinical practice, comprised of a diverse group of healthcare professionals. This included 16 psychologists, 8 social workers, 8 physiotherapists, typically 1 to 2 psychiatrists (though there are currently vacancies which have not been filled due to lack of availability of this professional group), 4 nurses, and 6 secretaries, as well as administrative staff and management. The composition of the multidisciplinary team includes the presence of members with foreign backgrounds (app. 20%).

### 2.2 Legal and ethical approval

The development of the contract for the collaboration on the DTD required 2 years of extensive legal work due to the intricate legalities needed to secure data across Danish regions. With this work completed, the DTD project rigorously protects patient data, adhering to the General Data Protection Regulation (GDPR) standards to ensure secure and confidential administration of data (Thøgersen et al., 2023). This emphasis on data security is essential for building trust with refugees with PTSD, who may have heightened vulnerability and mistrust. A comprehensive legal framework, developed with the assistance of a team of lawyers and GDPR experts, supports the ethical handling of data. This framework includes detailed procedures for data collection, storage, and patient consent, ensuring the protection of patients’ rights (Thøgersen et al., 2023). Patients are verbally informed about data collection by clinicians and provide consent via a written form containing all pertinent details. The current study data collection on the experiences with the training program was based on informed consent and stored according to the hospital’s ethics approval system license number OP_1964. We stored data in the OPEN (Open Patient data Explorative Network, Odense University Hospital, Region of Southern Denmark).

### 2.3 Training program

The training program involved several activities (kick off workshop, work groups, dialog forums, skills training workshop, small practice groups, and larger practice groups) planned and carried out in a period of almost 2 years (October 2021–August 2023). See Table 1 for an overview of activities. The project was structured to facilitate continuous development of training activities with a small project group comprising an administrative staff member, the project manager, and the principal investigator. Throughout the project period this group consistently worked to plan the next activities based on response and feedback from clinicians. This iterative process intended to engage clinicians in making improvements to the training process. The training period from first kick off to full implementation in the clinic lasted short of 2 years, while the originally intended duration was 1 year. This prolongation was due to the delay in legal approvals for the database collaboration.

**Table 1:** Training program.

	Activity	Time	Output
1.	Kick off workshop (3 h) with all clinicians together (first group discussion)	October 2021	A summary sheet from all groups
2.	Work group addressing adaptation of the database to clinical work procedures (3 h)	March 2022	Written work procedure
3.	Work group developing e-learning and information to patients and interpreters (several meetings)	April 2022 – August 2022	E-learning program
4.	Peer-led discussion of the database and implications for routine clinical practice at staff-management meetings, and patient-management meetings (several meetings)	April 2022	Share experiences, tips, and best practices
5.	Kick off first follow up workshop with all clinicians together (3 h) (second group discussion)	April 2022	A summary sheet from all groups
6.	Training workshop (introduction to the electronic system, and experimenting and role playing to develop skills and competency) (6 h in two groups)^*^	August 2022	All questions collected in document
7.	Follow up workshop (1 h) with all clinicians together	December 2022	All non-resolved questions collected
8.	Work group developing a clinical guideline for using the PROM data to plan and monitor treatment within the daily clinical practice	February 2023–June 2023	A written clinical guideline including conference structure utilizing the PROM data
9.	Small group of clinicians practice using the database with their real patients	February 2023 – March 2023	Experiences collected in a questionnaire
10.	Larger group of clinicians practice using the database with their real patients	April 2023 – June 2023	Experiences collected in a questionnaire
11.	Kick off second follow up workshop (2 h) with all clinicians together (third group discussion)	June 2023	A summary sheet from all groups
12.	Psychologist role play practice 6 h	August 2023	Na.

^*^Usage of the database for patient interactions was suspended from August to December 2022, owing to pending resolutions regarding the General Data Protection Regulation (GDPR) concerns.

### 2.4 Material for evaluation

To evaluate the training, we employed two data sources. The first was the collection of handwritten summary sheets from the kick-off workshops. These sheets were the result of group discussions among all staff members, focusing on three key questions: *What are your hopes for DTD? What are your concerns regarding DTD?* and *How do you envision data being utilized in patient treatment?* This approach provided insights into the staff’s’ perspectives, expectations, and concerns about the initiative, contributing to a comprehensive understanding of its potential impact.

Secondly, we invited all clinicians who had participated in PDSA processes and used the database with their real patients to take part in focus group interviews. This amounted to 18 clinicians, and 15 clinicians across three focus groups participated. The participants, representing the clinic’s staff composition, included two secretaries, two nurses, two physiotherapists, three social workers, and six psychologists. Notably, due to the absence of doctors among staff members during the interview period, this profession was not represented in the participant sample.

The objective of the focus group interviews was to assess how well the training program equipped clinicians for utilizing the electronic database in regular clinical practice. This evaluation was structured around three key themes: (a) examining how hands-on learning improves skills, (b) exploring clinicians’ attitudes toward integrating technology into patient care, and (c) understanding clinicians’ perspectives on the use of PROMs in patient treatment. With these themes as a foundation, we crafted a semi-structured interview guide to encourage an open conversation focused on the clinicians’ experiences and reflections on the training, as well as their making meaning of technology and PROMs in their clinical practice. Conducting focus group interviews allowed clinicians to share and reflect on their experiences and insights together. This group format fostered the exchange of diverse viewpoints and the discovery of common experiences, enriching the overall analysis by revealing how opinions are formed in a group context. We synthesized the findings from the group discussion with the findings from the focus group interviews in the Discussion.

### 2.5 Data analyses

As we reflect on our roles and their impact on our analyses, we, as a psychologist and an anthropologist, consider ourselves external to the group being studied. Despite the first author’s background in psychology, which aligns with the primary training of most participants, our different work orientations and tasks set us apart. This external perspective permits a detached analysis of the study, enabling an observation of the group’s dynamics and practices without direct involvement. We recognize our favorable stance toward both PROM and technology and acknowledge that the overarching goal of our research was the successful implementation of the database. Our philosophical approach aligns with critical realism, and our epistemological standpoint is contextualism. Our research interest was concentrated on an experiential perspective, aiming to delve into the meanings, views, perspectives, experiences, and practices expressed by the group being studied.

#### 2.5.1 Group discussions

We processed the data derived from the repeated data points by initially transcribing all text from the presentation materials onto a computer, categorizing it according to the questions posed (hopes, concerns, and clinical application), and the time of data collection (October 2021, April 2022, and April 2023). Subsequently, we applied coding to all the entries under each question and organized these codes into thematic categories. We discussed and refined the codes and thematic categories. Each theme from every data point was examined, and we explored the interconnections between themes across the three data points.

#### 2.5.2 Focus group interviews

The second author (LK) moderated the three focus groups, and the project coordinator at the clinic served as an observer, capturing group dynamics and other nuances that the dictaphone recordings missed. After completing the focus group interviews, the moderator (LK), the observer, and the first author (SBM) met to discuss the overall impressions and observations. This meeting aimed to develop a preliminary understanding of the data collected.

The focus group interviews were transcribed using NVivo 12 software. We adopted an inductive coding strategy for all transcripts. Our analysis was guided by a reflexive approach to Thematic Analysis (TA) (Braun and Clarke, 2022; Braun et al., 2023), emphasizing the importance of reflexivity throughout the analytical process to explore patterns of meaning across the focus group datasets. TA is recognized for its theoretical adaptability in analyzing qualitative data and follows a six-phase, practical analysis. The first phase of this six-step model involved immersing ourselves in the data through multiple readings. The transcription of the three focus group interviews was done by one of us (LK), and both of us reviewed and then discussed these transcripts. The second phase entailed generating initial codes, undertaken by LK, with a focus on descriptive, semantic coding. This phase included refining the codes by reviewing, editing, deleting, or renaming them. In the third phase, these codes were grouped into broader thematic clusters, followed by a collaborative discussion between us to refine and finalize the themes. We did not pursue coding agreement or reliability; instead, our discussions served as a collaborative and inspirational platform for our analysis. In the fifth phase, themes were defined, named, and collaboratively integrated into a thematic map. The conclusive sixth phase encompassed the composition of the final analysis.

### 2.6 Theoretical framework

In our pursuit of comprehending the integration of technology into the clinical practices of our clinicians, we employ a theoretical framework rooted in postphenomenology to the discussion of our findings. Postphenomenology, as developed by scholars like Don Ihde, builds upon the foundations of phenomenology but redirects its focus toward the complex interplay between technology and human existence (Ihde, 1990). While phenomenology seeks to understand the essential nature of phenomena, postphenomenology investigates how technology mediates and shapes these very phenomena (Ihde, 2023). Postphenomenology provides a unique lens through which we can explore and comprehend the complex relationship between human actors and technology.

## 3 Analyses

### 3.1 Group discussions

From the analysis of the group discussions, we generated key themes regarding clinicians’ hopes for technology implementation in clinical practice. These themes include clinical insight, research collaboration, political impact, successful implementation, systematic approach, and role clarity, as detailed in Table 2. In the context of clinicians’ concerns regarding the implementation, we generated the following themes: data dread, research dominance, resource challenge, vulnerable patient population and technology disconnection (see Table 3). Lastly, from the during group discussions on using data in patient treatment, we generated three predominant themes: evidence-based treatment, personalized treatment, and policy impact (see Table 4).

**Table 2:** Group discussion – hopes.

Question for group discussions	Themes identified based on group discussions	Analysis of group discussion 1 October 2021	Analysis of group discussion 2 April 2022	Analysis of group discussion 3 April 2023
What are your hopes for DTD?	Clinical insight	Clinicians hope to determine how to provide the best treatment for the patients by monitoring the treatment based on evidence-based measurements.	Clinicians hope for an increased focus on the benefit of evidence-based treatment and on the patient’s perspective in relation to the clinical effect.	Clinicians hope for an increased focus on the benefit of evidence-based treatment and on the patient’s perspective in relation to the clinical effect.
	Research collaboration	Clinicians hope for national collaboration that will contribute with knowledge of international scope for the benefit of the patients.	Clinicians hope to contribute to research in international contexts and to share knowledge nationally across clinics.	Clinicians hope to gain advantage of collaborating with researchers – both parts get better informed.
	Political impact	Clinicians hope that better understanding the patient target group will enhance focus on their needs across various sectors and in the political sphere.	Clinicians hope that better understanding the patient target group will enhance focus on their needs across various sectors and in the political sphere.	Not in focus.
	Successful implementation	Clinicians hope to ensure a successful implementation throughout the entire department.	Clinicians hope that making changes to the workflow as needed throughout the process will ensure successful implementation.	Clinicians hope that successfully implementing DTD will improve the approach to patient care.
	Systematic approach	Not in focus.	Clinicians hope for improved structure and systematization.	Clinicians hope that a systematic data approach will be beneficial for the patients.
	Role clarity	Not in focus.	Not in focus.	Clinicians hope for improved focus on skill development, teamwork, and role clarity among professional groups

**Table 3:** Group discussions – concerns.

Question for group discussions	Themes identified based on group discussions	Analysis of Group discussion 1 October 2021	Analysis of Group discussion 2 April 2022	Analysis of Group discussion 3 April 2023
What are your concerns for DTD?	Data dread	Clinicians fear facing the unfamiliar and failing in gathering data in clinical practice.	Clinicians fear collecting data that will not be used in clinical practice.	Clinicians fear collecting data that will not be used in clinical practice.
	Research dominance	Clinicians fear that research will dominate practical implementation.	Not in focus.	Clinicians are concerned that the vulnerability of the patient population may compromise data validity (repeated in “Vulnerable patient population.”)
	Resource challenge	Clinicians worry about the high demands on resources, both human and financial, in meeting external requirements.	Clinicians worry about the high demands on resources, both human and financial, in meeting external requirements.	Clinicians are concerned about the practicality of implementation and whether it is a worthwhile use of resources for both patients and clinicians.
	Vulnerable patient population	Clinicians fear that the patient population is too vulnerable to participate in the data collection.	Clinicians worry that the vulnerable patient population might not gain sufficient benefits from participating in data collection.	Clinicians are concerned that the vulnerability of the patient population may compromise data validity.
	Technology disconnection	Not in focus.	Not in focus.	Clinicians fear that the use of technology will negatively impact the contact and alliance with the patient.

**Table 4:** Group discussions – clinical application.

Question for group discussions	Themes identified based on group discussions	Analysis of group discussion 1 October 2021	Analysis of group discussion 2 April 2022	Analysis of group discussion 3 April 2023
How do you imagine data being used in patient treatment?	Evidence-based treatment	na	Clinicians believe that data collection will support evidence-based treatment, leading to better treatment outcomes.	Clinicians foresee that feedback between research data and clinical application will enhance evidence-based practice, both now and in the future.
	Personalized treatment	na	Clinicians envision that data collection will aid in creating personalized treatment plans for each patient.	Clinicians envision that data collection will aid in creating personalized treatment plans for each patient.
	Policy impact	na	Clinicians anticipate that information will be accessible and useful to relatives and decision makers, both currently and in the future.	Clinicians envision that data collection should serve political objectives to enhance opportunities for the target population.

### 3.2 Focus groups

**Figure 1:**
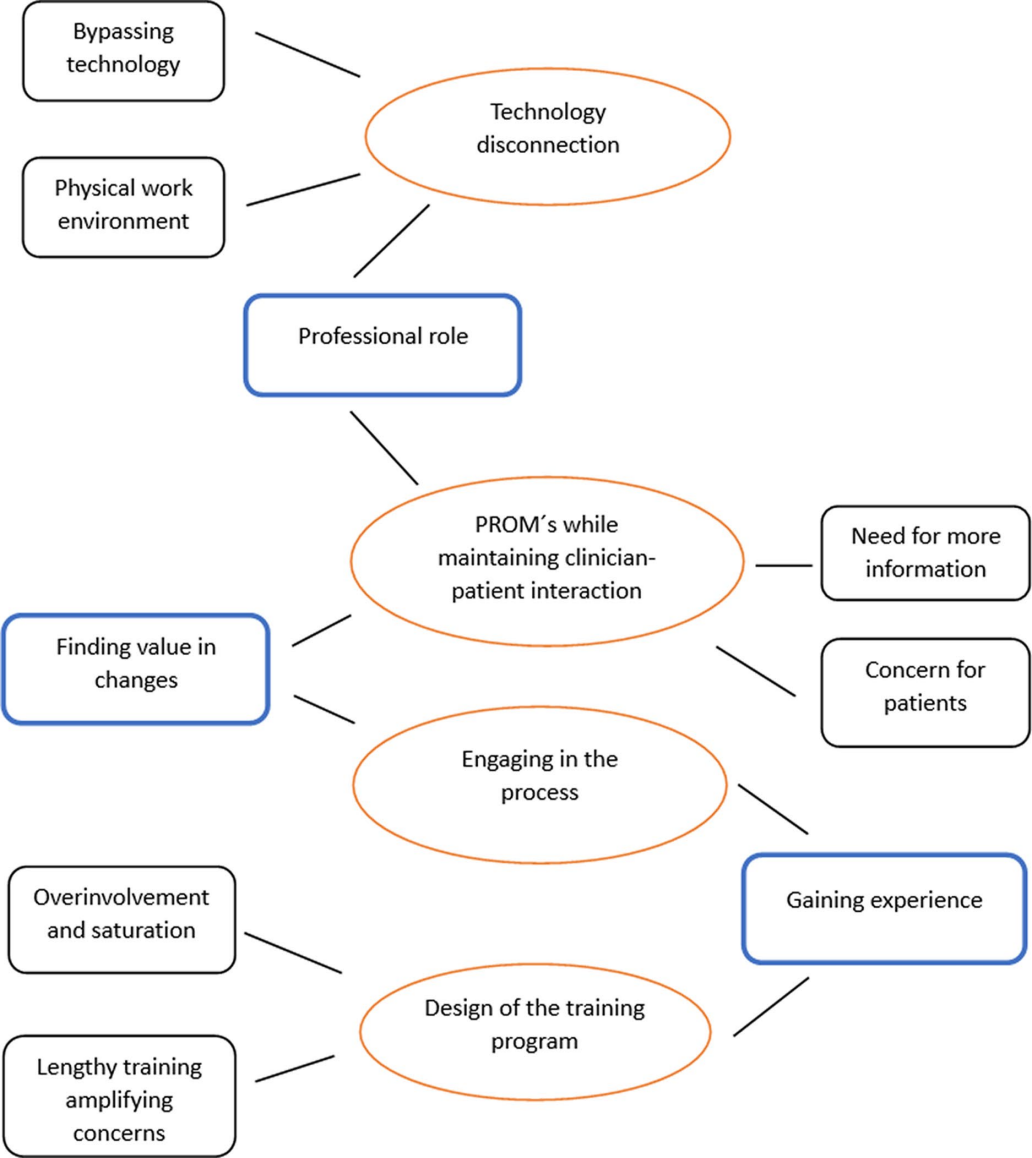
Thematic map.

From the focus group analysis, we generated four main themes that were emphasized and discussed across all three focus groups, receiving broad endorsement from the clinicians: technology disconnection, PROM’s while maintaining clinician–patient interaction, design of the training program, and engaging in the process. Figure 1 presents the thematic map showing the interconnections between these themes.

#### 3.2.1 Technology disconnection and PROM’s while maintaining clinician–patient interaction

We found that clinicians employed multifaceted attitudes and strategies in response to the integration of technology – the use of computers and PROMs – into clinical practice. However, a prevalent perception existed that the use of technology would influence the interaction between clinicians and patients, consequently giving rise to concerns regarding patient experience.

Despite instructions to directly use computers for entering PROM data during patient contact, clinicians often adopted alternative strategies to reduce or bypass computer use in patient interactions. One prevalent approach involved printing assessments on paper, a practice aimed at preserving the quality of the clinician–patient interaction. Clinicians talked about their concerns about the computer creating a barrier between themselves and their patients, as “a screen between me and the patient.” Furthermore, using paper assessment served the purpose of allowing clinicians to include additional information they considered necessary for the patient’s record beyond the structured assessment information from the DTD. Additionally, clinicians found that the interaction with the patient was negatively affected when they focused on conducting assessments without allowing their patients more spontaneously to provide further context in a more unstructured way as they would usually do. Some clinicians also talked about challenges associated with adapting their physical working environment to accommodate both computer use and patient communication. The discussions presented variations among clinicians in the attitudes toward using computers in their interactions with patients. Some clinicians reported being “unafraid of technology,” while others expressed having or having had significant resistance to using technology in patient encounters due to the above-mentioned concern for the quality of the clinician–patient interaction. Interestingly, our findings suggested variations in clinicians’ receptiveness to integrating technology, depending on their professional group. This appears to be linked to their own views of their professional roles. In particular, nurses were more at ease with the introduction of computers into consultation rooms, viewing this as a relatively straightforward and unproblematic practice.

*“I am used to using the computer during my sessions. […] For me, it’s just the computer standing next to me, and then the patient.”*

*Nurse 1.*

*“I do not feel like there’s any distance from the patient because often, I’m still in front of my computer when I have sessions with patients. I can look up information about medication and things like that while we are talking.”*

*Nurse 2.*

In contrast, especially psychologists expressed dissatisfaction with the use of technology during clinical sessions. They perceived that the presence of the computer adversely affected the quality of the interaction with the patient:

*“I constantly need to elaborate, like ‘you see, this is because it’s the diagnostic assessment, and it’s not the same as when your treatment begins.’ And ‘I’m so sorry, I’m listening while you are talking.’ And then I look up, and then I tap into the computer. Oh, it does not come as smoothly. I do not have that eye contact.”*

*Psychologist 2.*

*“I could also do without the computer. I do not think either that it was very pleasant. I also felt that the interaction was being affected. And the patient I had [in this session] was actually pretty resourceful. However, I just heard myself coming up with explanations for why I brought the computer [into the session], and that ‘I am still here, but I just had to write on the computer’. And ‘now we put it away’ and ‘now we bring it back again’.”*

*Psychologist 5.*

The statements indicate that psychologists prioritize their patient relationships in clinical settings and believe that technology interferes with the therapeutic process. Clinicians are deeply worried about technology’s potential to affect their empathy toward patients. By clarifying the purpose of using computers and reassuring patients of their commitment to remaining engaged, they aim to prevent technology from becoming a barrier or weakening the crucial therapeutic bond essential for effective therapy. Psychologist 5’s comment about the patient being “pretty resourceful” reflects an appreciation for the patient’s capability to handle technological disruptions. This perspective appears to stem from dealing with a patient who has more resources than the typical patient.

Psychologists expressed feeling self-conscious about the disturbance caused by using computers and tried different methods to lessen its adverse effects. For instance, they often justified their computer use during patient interactions, making excuses or explaining the need to bring it into the session, and regularly alternated between using the computer and setting it aside. These insights highlight the complex task of integrating technology into clinical practice, balancing its use while maintaining an effective therapeutic connection, a challenge also reflected in statements from a social worker:

*Social worker 2: And I do not think I’d be able to figure out how not to have the assessments with me [printed in paper] and take notes while we are sitting there. So, I’m still in the process of how I do it, actually. I have not found a way into it yet, whether I start with the assessments or if we take it a bit more loosely. Also, because some of the questions, like isolation, I can remember, we need to inquire about, it can naturally come up in a conversation or generate something natural to talk about based on what I’ve asked. So, it does not have to be as mechanical as I initially thought when I first sat there. So, it’s also a process. It becomes part of your working style in how we do it. Interviewer: So, A. Can you say that your attitude toward having to bring the computer and use assessments has changed positively as you have had the opportunity to try it out?*

*Social worker 2: Yes, it occupies less space in my mind. It’s less anxiety-inducing than it was initially. Because back then, I could also have this feeling, like, I’m more of a pen-and-paper kind of person, so how much should this screen really dominate the conversation. But it can take up less space now than it could at the beginning. And I think that’s a positive thing. Because I’m more natural about it, we just need to get the answer to this, dut dut, so we find it. And then there may be a conversation about it, maybe not. But then it can go away again.*

The quotes provided by Social Worker 2 offers a detailed insight into the practical use of the technology, highlighting both the challenges faced and how they were overcome. Initially, the social worker was hesitant about integrating the technology into her workflow. The main concern was the transition from a traditional pen-and-paper approach to a digital one. This reflects a common concern among clinicians who may feel more comfortable with conventional methods and perceive new technologies as disruptive or impersonal. However, with increased familiarity, the social worker began to see technology as not inherently inflexible, eventually adopting a more positive view and recognizing its potential to facilitate natural and meaningful patient interactions. This mental adjustment is essential as it indicates a shift from viewing technology as an obstacle to seeing it as a supportive tool. This case exemplifies the common trajectory from initial caution to eventual acceptance and integration of technology in clinical settings. By demonstrating flexibility and openness to trial and error, Social Worker 2 effectively incorporated the technology into her practice, enhancing her ability to maintain meaningful clinician–patient interactions while utilizing new technologies. This underscores the importance of personalized adaptation of strategies to effectively integrate technology into healthcare practice in a way that protects the clinician–patient relationship.

#### 3.2.2 Design of the training program and engaging in the process

Our findings indicate that the duration of training impacted clinicians’ attitudes, competencies, and readiness for change in various ways. Many clinicians experienced that the training was excessively long, leading to unnecessary concerns:

*Psychologist 4: “For me, this whole level of activity has been way, way, way too long. It’s like using a sledgehammer to crack a nut. We’re exhausted in the process even before we get started. I mean, we have not even reached the starting line yet, and it feels like we have run an ultramarathon, in my opinion. With all the discussions, implementation, and all that talk. And all those working groups, and all those value streams, and all that blah, blah, blah. So, for me, and it may just be my personal standpoint, we should have had 3 months, boom, then we are up and running. I’m simply already exhausted before we even get started. I was already feeling this way before I even joined the second pilot testing. That’s why chose to say, ‘now you are going to join the second pilot testing, because otherwise, you’ll end up completely out of it if you do not get involved yourself’.”*

*Interviewer: What was supposed to happen in those 3 months?*

*Psychologist 4: Introduce me to the test batteries I need, what I should do with them, and why they make sense in the end. It would have been fine if I had just received an introduction to the database, and then we could go.*

*Interviewer: So, what about the work procedures?*

*Psychologist 4: That’s just something that someone needs to decide how they should be. And then I’ll do what I’m told needs to be done. There have been too many cooks spoiling the broth, in my opinion.*

The quote shows Psychologist 4’s view of the training program as overly long, advocating for a substantial reduction in duration. She emphasized the need for straightforward guidelines and a process where decisions on workflows are made at the organizational level, without requiring extensive input from clinical staff. However, opinions among clinicians varied. For example, a social worker, despite having a minor role in collecting clinical data via the DTD, stressed the importance of her involvement in the process:

*“Even though I do not have such a significant role, if there were something new, I find it interesting to stay updated on it. But it’s more because I think there have been repetitions without much new information. […] However, I think it was fine that we heard about it when we did. Because I think it’s good to be [involved], so you can have an influence on things.”*

*Social worker 1.*

The quote suggests that while the social worker values her involvement, she felt that there were excessive repetitions of status updates on the implementation in clinic meetings, often with little new information.

Regarding the prolonged training program and its impact on clinicians, psychologist 4 expressed:

*Psychologist 4: Well, it’s my perspective, and it does not mean that I would not have had any opinions if it had only taken 3 months. But for me, I think we would have avoided a lot of anxiety among the clinicians about stepping into this if we had not emphasized it as much as we have.*

*Interviewer: Can you describe the anxiety?*

*Psychologist 4: Well, there has been a lot of resistance to starting this in the first place. And I think that resistance has actually only been reinforced by this long process.*

*Interviewer: And what did the resistance initially revolve around?*

*Psychologist 4: I do not know because I did not have much resistance to it. But there were just many people wondering, ‘uh, what are we supposed to do’, and ‘what are we being thrown into?’*

The psychologist believed that too much emphasis on the procedural aspects increased anxiety, indicating the need for a structured and confined approach to preparing clinicians for technology use in their practice. An excessively long process might cause unnecessary concerns. The quote emphasizes the need to balance adequate preparation with the risk that prolonged attention could heighten, rather than alleviate, anxiety and resistance. Psychologist 2, however, viewed the extended training duration differently, perceiving it as a maturing process that improved her competencies and readiness for upcoming changes:

*“I think it has contributed to my maturation and readiness for it. It certainly does not feel like something that happened overnight. […] It has been fine that it has been based on the idea of ‘let us try things out now.’ So, I actually feel sincerely that I have been part of a pilot testing. Where it has also been okay to gain experiences, okay to make mistakes, or feel that you are not good enough at it yet. So, I think it has contributed to maturing in a positive way.”*

*Psychologist 2.*

Despite the extended duration of the training program, the psychologist maintained a positive attitude. The phrase “does not feel like something that happened overnight” highlights that the psychologist recognized that learning and development take time. Such contrasting views of the impact of the length of the training program underscore the complex link between training duration and variability in clinicians’ needs.

Further, the psychologist in the quote above appreciated the training program’s experimental approach, emphasizing the importance of trying things out. This perspective is consistent among all clinicians who participated in the focus groups, and it reflects their enthusiasm for the PDSA pilot processes. Some of the clinicians expressed how they were eager to begin the pilot testing to gain firsthand experiences and prevent concerns from escalating further. They wanted to put their concerns to the test and determine whether integrating technology into clinical practice was as challenging as they were being perceived:

*“I feel that some of the discussions we had in the beginning, they contributed to confusing me. And maybe also making it a little more negative than it perhaps is. […] I think some of the discussions that have taken place have been very much about concerns and ‘that’s not good’, and ‘oh, we cannot do that’. It has actually affected me. Even though I’ve tried to say… I did agree to participate in the pilot testing because ‘how bad can it be?’ I mean, sorry. That’s actually how I think, and I would like to find out that for myself.”*

*Physiotherapist 1.*

The physiotherapist pointed out that the training program’s discussions often focused on concerns and resistance to changes, negatively affecting her. Nonetheless, she chose to join the pilot testing to experience it directly. Her remark, “how bad can it be?,” indicates an openness to trying out the changes and personally evaluating their effects.

Also, on the notion of trying something firsthand rather than speculating about it, a social worker emphasized the importance of engaging in the actual pilot testing allowing the clinicians to form informed opinions:

*“I can also think back 6 months, where I thought, ‘okay, let us just try it’. Instead of talking about it, let us try it. Everyone. Because then we have something to discuss, as everyone can have an opinion about all sorts of things. Otherwise, it becomes like social media, where I complain about something I know nothing about. Let us try it so we have something real to base it on.”*

*Social worker 1.*

The social worker drew a comparison with social media, where people often express strong opinions without substantial knowledge or firsthand experience. The quote suggested that firsthand experience empowered the clinicians to contribute meaningfully to discussions. Also highlighting clinicians’ appreciation for hands-on experience during training is expressed in the following exchange involving two psychologists (Psychologist 4 and Psychologist 5) and two social workers (Social worker 2 and Social worker 3).

*“Psychologist 5: So for me, it’s those three things: It’s the visit from the collaborators, the explanation for why this makes sense, that we together are six clinics and so on. And then I think that it’s the first and second pilot testing, where we actually get our hands on it and are allowed to dig in and say, okay, how can this be done, and what are those specific words that I need to make sure to formulate in a certain way, or how does it work when the internet breaks down,* etc.

*Social worker 2: I completely agree. It’s the pilot testing that have made a difference for me.*

*Interviewer: What was good about the pilot testing?*

*Psychologist 4: Getting our hands on it, instead of just sitting and talking about it.*

*Interviewer: You both nod, [Social worker 2] and [Social worker 3].*

*Psychologist 4: And finding out that maybe there’s something that’s okay, it went smoother than I thought, or there was something else that was harder than I thought. Finding that out is the first step.*

*Social worker 2: I also thought it was nice when we sat, and just tried it out. It was the first time to log on to the database and check out the questions there are there, and so on. That was the first, now I say physical meeting with the database. I actually thought that was nice. Then we could just see, what is this, this thing. That monkey we talk about all the time…”*

In this dialog clinicians stressed the importance of practical engagement either through role playing with a colleague to gain first experiences or through the pilot testing with real patients. This approach enabled them to better understand and navigate the tools used in their clinical practice, uncovering real-world challenges and benefits, and gain confidence to proceed. Overall, these insights underline the significance of experiential learning in training for effective technology adoption in clinical settings.

## 4 Discussion

This study was conducted to evaluate a training program aiming to familiarize clinicians with new technology for patient care. Specifically, it targeted clinicians who were initially inexperienced with integrating structured, data-driven assessments through an electronic database. The objective was to actively involve clinicians in the learning process, encouraging them to share their concerns and suggestions about the technology. This feedback mechanism was designed to enhance the training process, ultimately fostering a productive relationship between the clinicians and the technology.

Clinicians in this study showed a deep commitment to their refugee patient group, focusing on patient needs and perspectives, aligning with principles of social justice. This dedication influenced their hesitation to integrate the new technology with PROM. Especially, clinicians express concerns about the potential negative influence of technology integration on the patient-therapist relationship. It is crucial to further address this aspect in the management of technology-based treatment settings, ensuring sustained engagement from both patients and therapists (Hoffmann et al., 2020).

Within Don Ihde’s post-phenomenological framework, (Ihde, 2023) the successful integration of technology into practice is contingent upon its embodiment, wherein it becomes an extension of the human body that changes our experiences. For example, glasses, once worn, become part of the wearer, enhancing vision and acting as a mediator with the environment. However, new or unfamiliar technology can challenge this relationship, as seen with clinicians whose traditional patient interactions were disrupted. Ihde (2023) suggests that technology should extend beyond being just an external tool, becoming an integrated aspect of our bodily experience and altering our perception of the world. Yet, in this case, clinicians did not seamlessly integrate the new technology into their practice as an extension of themselves but rather saw it as more of a hindrance than a help. This uncertainty to embody the technology may stem from it being imposed rather than chosen by the clinicians themselves. Despite management and researchers encouraging clinician involvement in the implementation process and emphasizing the technology’s potential to meet patient needs and attract desired political attention, the transition from conventional practices for interaction with the patients to new methods integrating technology was met with some hesitancy. Clinicians found the transition of patient interactions into digital data, which involves a hermeneutic relationship where technology interprets or changes information, to be unfamiliar and invalid. This situation is comparable to replacing direct experience of the weather with the use of a thermometer. Just as a person might initially distrust a thermometer’s reading, preferring to rely on their direct sensory experience to estimate temperature, clinicians faced a similar challenge. They needed to shift from their established, direct methods of patient interaction to trusting in a digital representation of these interactions for understanding the patient. This shift requires a deliberate effort to trust the technology’s interpretation, understanding that it is a new form of perceiving and interacting with patient information, just as a thermometer offers a new way of understanding the weather. Embracing this change demands not only a trust in the technology but also an adaptation of one’s professional practice to incorporate this new form of mediated understanding.

The challenges in familiarizing clinicians to the new technology, which resulted in their cautious approach and an unrealized opportunity for experiencing both embodiment and a hermeneutic relationship with the technology, seemed shaped by their commitment to safeguarding patient relationships and maintaining established professional roles. Clinicians predominantly viewed patients as vulnerable and felt an obligation to protect them by maintaining a secure and validating relationship. This viewpoint often positioned the technology as contrary to their primary objective of ensuring patient well-being, thereby creating a conflict for clinicians between providing optimal patient care and complying with management directives. This complex dynamic underscores the nuanced challenges in integrating new technologies into established healthcare practices. Despite the overall cautious approach toward the new technology, it is important to highlight that some clinicians reported having positive experiences with it. This suggests that under certain conditions, the technology has the potential to be successfully integrated into clinical practice. These positive experiences could be attributed to various factors such as specific personal attitudes of the clinicians, the nature of their patient interactions due to differences in professional roles, or even the particular ways in which they used the technology.

When designing the training, our primary aim was to establish a process that would alleviate concerns and negative perceptions about the technology. This was achieved by creating a supportive environment where clinicians could openly discuss and shape future workflows involving the technology. Additionally, we incorporated experimental learning and pilot testing, allowing clinicians to explore and familiarize themselves with the technology in a variety of ways. Unfortunately, the training process, which extended beyond its planned duration, had unforeseen consequences. The extended discussions in the training, combined with insufficient practical experience, appeared to exacerbate rather than mitigate the clinicians’ pre-existing concerns and tendencies to avoid the new technology. Instead of clarifying the technology’s features and addressing fears and negative attitudes, this approach ended up reinforcing them. This phenomenon can be interpreted using Don Ihde’s concept of multistability, which asserts that the interpretation of technology is influenced by individual perspectives, shaped by existing knowledge and experience (Ihde, 2023). According to this concept, an object or perception can be interpreted in multiple ways, with each interpretation being equally valid, depending on one’s prior knowledge and familiarity. Consequently, certain understandings of technology might remain elusive until they become culturally ingrained. In our training context, clinicians’ commitment to patient-centered care and their professional culture likely influenced their initial perceptions of the technology. This predisposition meant that discussions often solidified their initial views instead of introducing them to the technology’s possible benefits. This situation created a cycle where the established viewpoints of the clinicians, shaped by their professional culture and patient care approach, dominated. This dominance made it difficult for them to perceive the technology in a new light, thereby impeding the adoption of novel patient interaction techniques.

This scenario can be maintained through avoidance behavior, where actions deemed unpleasant are avoided unless the long-term benefits are clearly understood to outweigh the immediate discomfort. In our training context, the delay in the process may have inadvertently contributed to the clinicians’ avoidance behavior, as extended discussions occurred without corresponding changes in behavior. This aligns with the Temporal Self-Regulation Theory (Hall and Fong, 2007), which suggests that people’s decisions are often influenced more by immediate costs and benefits rather than long-term outcomes. As a result, a tendency to favor short-term comfort over long-term gains can lead to avoidance behavior. It’s important to acknowledge, though, that this cautious approach also reflects the clinicians’ strong dedication to patient welfare and their careful consideration of new practices. With the potential long-term implications of the training program and technology integration in clinical practice in mind, it is imperative to study impacts of the training program over time. This longitudinal approach in a follow up study is crucial for gaining insights into the enduring effects and evolving dynamics associated with the implementation of web-based PROMs in clinical settings.

## 5 Limitations

The observed increase in concern from the first to the third analysis of group discussions which included all staff members could be indicative of the predominate perspective from across the clinic. It is also possible that some clinicians may have been reserved in sharing their complete views, possibly due to concerns about potential outcomes. A notable limitation of the data from the focus group interviews is that it primarily reflects the perspectives of clinicians who willingly participated in the PDSA processes. These clinicians may have been more open to technology implementation from the outset. Additionally, incorporating qualitative insights from management and patients could have provided valuable perspectives. In subsequent studies, the incorporation of user feedback from refugees is crucial as it offers invaluable insights into the usability and effectiveness of web-based PROMs, contributing significantly to optimizing patient care. Furthermore, having the resources to conduct individual interviews might have uncovered insights not revealed in the focus group settings.

## 6 Recommendation for future training programs

Based on our experience with the current training program, we advise a revised strategy for future initiatives that prioritizes early and direct engagement of clinicians in using technologies such as computers and PROMs in patient care. The use of numerous small PDSA cycles involving clinicians in various tasks did not fully yield desired results, possibly due to a delayed ‘Do’ phase. Successful technology implementation in clinical settings hinges on integration with workflows, respecting clinicians’ judgment, and demonstrating clear patient care benefits. We advise future training programs to involve clinicians directly from the start, offering hands-on experience with the technology. Transitioning from extensive planning to practical application, like using role plays to visualize technology integration into workflows, is crucial. This ensures clinicians gain firsthand experience with the technology before considering its integration into workflows. Alignment with existing workflows is crucial for effective technology adoption. Additionally, acknowledging the unique characteristics of different clinician groups, including their openness to change, technological readiness, and specific approaches to patient care, is essential. By customizing strategies to address these individual differences, the effectiveness of integrating new technology can likely be enhanced. Key Recommendations Include:

- Positive Narratives: Focus training on both the immediate and long-term advantages of the new technology, demonstrating how it can simplify daily tasks, improve patient-clinician interactions, and enhance care quality in the short term. This could be conducted through clinical ambassador peers presenting real case stories with good outcome.
- Minimizing Disruption for Clinicians: Introduce the technology gradually, starting with basic features and progressing to more complex ones. This can help clinicians adjust comfortably at their own pace, minimizing the sense of disruption.
- Tailored Training Modules: Create training modules tailored to the specific needs and concerns of different clinician groups, taking into account their unique professional cultures and patient care philosophies.
- Hands-On Training: Offer ample opportunities for clinicians to use the technology in controlled settings, allowing them to experiment and directly observe its impact receiving peer-support and positive feed-back.
- Reward Positive Engagement: Acknowledge and reward engaged clinicians who actively participate in the training and effectively integrate the new technology into their practice.

By focusing on these recommendations, training for new technology in healthcare can hopefully be better aligned with clinicians’ immediate needs and preferences, potentially leading to more meaningful integration into clinical practice.

## 7 Conclusion

In conclusion, the training program’s extended dialog and delayed hands-on implementation may have inadvertently strengthened clinicians’ existing reservations and hesitance toward adopting new technology, instead of modifying them. Interpreted through the lens of Don Ihde’s post- phenomenological philosophy of technology and the Temporal Self-Regulation Theory, it appears that clinicians were more influenced by immediate inconveniences than potential long-term benefits, particularly in their view of the technology’s impact on patient interactions. Yet, this cautious approach should be understood as a reflection of their strong dedication to patient care and a thoughtful approach to adopting new practices. This perspective is essential in shaping more nuanced and effective strategies for future technology training and integration within clinical settings. Our study underscores the complexities of clinician training and technology adoption in refugee healthcare settings, emphasizing the ongoing journey of adaptation and the necessity of clinician engagement for effective technology integration.

## Data availability statement

The datasets presented in this article are not readily available because the data is transcribed interviews. Requests to access the datasets should be directed to stinebm@health.sdu.dk.

## Ethics statement

Ethical approval was not required for the study involving humans because in Denmark, qualitative research does not require ethical approval from the National Committee on Health Research Ethics (www.nvk.dk). The study was conducted in accordance with the local legislation and institutional requirements. The participants provided their written informed consent to participate in focus group interviews.

## Author contributions

SM: Conceptualization, Formal analysis, Funding acquisition, Methodology, Project administration, Writing – original draft, Writing – review & editing. LK: Conceptualization, Formal analysis, Funding acquisition, Methodology, Project administration, Resources, Writing – review & editing.
